# Comparing Glycopyrronium/Formoterol Combination Therapy With Monotherapy in Moderate-to-Severe Chronic Obstructive Pulmonary Disease (COPD): A Narrative Review

**DOI:** 10.7759/cureus.58633

**Published:** 2024-04-20

**Authors:** Bollineni S Prada, Ulhas Jadhav, Babaji Ghewade, Pankaj Wagh, Ashwin Karnan, Anjana Ledwani

**Affiliations:** 1 Respiratory Medicine, Jawaharlal Nehru Medical College, Datta Meghe Institute of Higher Education and Research, Wardha, IND

**Keywords:** safety, efficacy, formoterol, glycopyrronium, combination therapy, copd

## Abstract

Chronic obstructive pulmonary disease (COPD) imposes a significant burden on individuals and healthcare systems globally. While bronchodilators, such as glycopyrronium and formoterol, are cornerstone therapies for COPD management, combining these agents has gained attention for potentially improving outcomes compared to monotherapy. This comprehensive review aims to assess the efficacy and safety of glycopyrronium/formoterol (GFF) combination therapy versus glycopyrronium monotherapy in patients with moderate-to-severe COPD. Through a systematic evaluation of clinical trials and real-world evidence, we analyze the impact of combination therapy on lung function, symptom control, exacerbation rates, and health-related quality of life (HRQoL). Furthermore, we examine the safety profile of combination therapy, including adverse cardiovascular and respiratory events. Comparative analyses with glycopyrronium monotherapy provide insights into the relative benefits and considerations for treatment selection. Factors influencing treatment choice and future directions in COPD management are also discussed. This review underscores the potential of combination therapy in optimizing COPD treatment outcomes and highlights areas for further research and clinical practice refinement.

## Introduction and background

Chronic obstructive pulmonary disease (COPD) is a respiratory condition characterized by airflow limitation, typically associated with chronic bronchitis and emphysema [1]. It is a major cause of morbidity and mortality worldwide, with a significant impact on the quality of life of affected individuals. COPD is primarily caused by long-term exposure to irritants such as cigarette smoke, air pollution, and occupational hazards [2]. COPD represents a substantial public health burden globally, with estimates suggesting it will become the third leading cause of death by 2030 [3]. The disease is characterized by persistent respiratory symptoms, including cough, sputum production, dyspnea (shortness of breath), and exacerbations, often worsening over time. These symptoms result from progressive airflow limitation due to inflammation and structural changes in the airways and lung parenchyma [2].

The management of COPD aims to alleviate symptoms, improve lung function, reduce exacerbation frequency, and enhance the overall quality of life for patients. While bronchodilators, such as long-acting muscarinic antagonists (LAMAs) and long-acting beta-2 agonists (LABAs), are mainstays of COPD treatment, combining these agents can offer synergistic effects by targeting different pathways involved in bronchoconstriction and airflow limitation. The rationale for combination therapy lies in the potential for enhanced bronchodilation, improved symptom control, and reduced exacerbation risk compared to monotherapy [1].

This review aims to comprehensively evaluate the comparative efficacy and safety of glycopyrronium/formoterol (GFF) combination therapy versus glycopyrronium monotherapy in patients with moderate-to-severe COPD. By synthesizing evidence from clinical trials and real-world studies, we seek to elucidate the advantages and limitations of combination therapy in managing COPD and offer insights into its optimal use in clinical practice. Additionally, this review aims to identify gaps in current knowledge and highlight areas for future research to optimize COPD treatment strategies further.

## Review

Mechanism of action and pharmacokinetics

Glycopyrronium

Glycopyrronium, or glycopyrrolate, is an anticholinergic medication characterized by its synthetic quaternary amine structure incorporating pyridine and a cyclopentane moiety [4]. Primarily utilized as a preoperative drug, it effectively inhibits salivary gland and respiratory secretions [4]. The predominant purposes for administering anticholinergics include inducing a sialagogue effect, promoting sedation and amnesia, and averting reflex bradycardia [4]. Glycopyrronium is one of the most frequently prescribed anticholinergic drugs [4]. Its mechanism of action entails the blockade of a specific natural substance responsible for stimulating sweat gland activity, rendering it efficacious in treating hyperhidrosis, particularly excessive underarm sweating [5]. As a muscarinic antagonist, glycopyrronium exhibits the highest affinity for M1 receptors, followed by M3, M2/M4, and M5 receptors [6]. Muscarinic receptors M1 through M4 are prevalent in the lung, with M3 primarily implicated in bronchoconstriction and airway secretions [6]. By antagonizing these receptors, glycopyrronium reduces the volume of respective secretions, thereby alleviating airway secretions observed in conditions such as COPD [4]. The medication is available in various formulations, including oral, intravenous, inhalation, topical, injection, and subcutaneous [6]. It holds approval for medical usage in the European Union and the United States and is listed on the World Health Organization's List of Essential Medicines [6]. Common adverse effects encompass dry mouth, urinary retention, headaches, vomiting, diarrhea, constipation, blurred vision, and urinary difficulties [6].

Formoterol

Formoterol, a long-acting beta-2 adrenergic receptor agonist, is a pivotal bronchodilator in managing asthma and COPD. Often employed in conjunction with other medications such as inhaled corticosteroids, it plays a crucial role in asthma treatment and bronchospasm prevention. Functionally, formoterol relaxes and widens the air passages within the lungs, facilitating easier breathing. Notably, it proves efficacious for the sustained maintenance treatment of airflow obstruction in COPD patients, encompassing chronic bronchitis and emphysema [7,8]. Regular utilization of formoterol can effectively reduce both the frequency and severity of asthma attacks. However, it is not designed to provide immediate relief during an ongoing asthma attack. Marketed under various brand names like Bevespi, Breztri, and Symbicort, among others, formoterol exerts its action on bronchial smooth muscle, resulting in rapid onset within 2-3 minutes and prolonged effects lasting up to 12 hours [8]. Administered via inhalation, formoterol acts swiftly to dilate and relax bronchial tubes, thereby mitigating symptoms such as coughing, dyspnea, and respiratory distress by augmenting airflow. Adherence to prescribed dosages and instructions is paramount to ensure the efficacy and safety of formoterol. Common side effects may include nervousness, headaches, muscle cramps, and nausea. However, vigilance is warranted as severe adverse reactions such as facial or throat swelling, breathing difficulties, chest pain, or rapid heartbeat necessitate prompt medical attention [9].

Combined Mechanism of Action

Glycopyrronium is a muscarinic antagonist with a notable affinity for M1 receptors, followed by M3, M2/M4, and M5 receptors. By impeding acetylcholine's influence on muscarinic receptors in the airway smooth muscle, glycopyrronium induces bronchodilation and diminishes secretions from salivary and sweat glands. This mechanism proves beneficial in managing conditions like COPD, as it reduces airway resistance and enhances lung function [10]. Conversely, formoterol functions as a LABA with rapid onset, stimulating intracellular adenyl cyclase to convert adenosine triphosphate (ATP) to cyclic adenosine monophosphate (AMP). Elevated cyclic AMP levels subsequently prompt the relaxation of bronchial smooth muscle, facilitating bronchodilation and augmenting airflow in conditions such as asthma and COPD [7]. When combined, glycopyrronium and formoterol synergistically complement each other's actions, offering a comprehensive approach to managing respiratory conditions. While glycopyrronium's anticholinergic properties target specific receptors implicated in bronchoconstriction and secretions, formoterol's beta-2 agonist activity enhances bronchodilation by fostering cyclic AMP production. This collaborative therapy provides an effective strategy for treating moderate-to-severe COPD by concurrently addressing bronchoconstriction and airway inflammation [11-13].

Efficacy of glycopyrronium/formoterol combination therapy

Clinical Trials Overview

Multiple clinical trials have investigated the efficacy and safety of GFF combination therapy in patients diagnosed with COPD [14]. These studies have consistently demonstrated that GFF is comparable to tiotropium/formoterol (TFF) in enhancing lung function and assessing health status [12,13]. Moreover, GFF and TFF have exhibited favorable safety profiles and tolerability among symptomatic COPD patients [12,13]. In a comparative study evaluating twice-daily glycopyrronium/formoterol (GF) versus once-daily glycopyrronium (G), GF emerged as more effective in enhancing lung function and alleviating symptoms in individuals diagnosed with moderate-to-severe COPD [14]. Another clinical trial compared the efficacy and safety of GFF with umeclidinium/vilanterol (UV) fixed-dose combination over 24 weeks, revealing that GFF was non-inferior to UV concerning lung function improvement and health status assessment [15]. Furthermore, a study assessing the efficacy and safety of a fixed-dose combination comprising fluticasone/formoterol/glycopyrronium (FFG) for COPD treatment demonstrated its effectiveness in enhancing lung function and alleviating symptoms among patients diagnosed with moderate-to-severe COPD [14].

Lung Function Improvement

Research indicates that GFF therapy is highly effective in enhancing lung function among individuals diagnosed with moderate-to-severe COPD [16]. Compared to alternative therapies such as budesonide/formoterol, GFF has shown superior efficacy in improving lung function, reducing the necessity for rescue medication and enhancing health-related quality of life (HRQoL) [16]. Moreover, GFF therapy has been associated with a decreased risk of exacerbations and clinically significant deterioration when compared to interventions such as glycopyrronium monotherapy and placebo [17]. In a direct comparison between twice-daily glycopyrronium/formoterol (GF) and once-daily glycopyrronium (G), GF demonstrated greater effectiveness in enhancing lung function and alleviating symptoms among COPD patients [18,19]. These findings underscore the substantial benefits of GFF therapy in improving lung function, alleviating symptoms, and reducing exacerbation risk in individuals with COPD. As a result, GFF emerges as a valuable and highly recommended treatment option for this patient population.

Symptom Control

Effective symptom management is pivotal in patient care, particularly in conditions such as COPD and palliative care settings. In Denmark, palliative care encompasses symptom control, nursing, and overall patient care when curative treatment is no longer feasible, with the primary goal of ensuring comfort and enhancing quality of life [20]. Moreover, specialized palliative care (SPC) emphasizes symptom control and end-of-life care, tailoring interventions based on gender, age, cancer diagnosis, and geographical location to optimize care delivery [21]. In the context of COPD, GFF therapy has demonstrated efficacy in enhancing lung function and alleviating symptoms among individuals with moderate-to-severe COPD [22]. This underscores the significance of implementing effective symptom management strategies in managing chronic respiratory conditions [22].

Health-Related Quality of Life Improvement

The GFF combination therapy has demonstrated substantial improvements in HRQoL among patients diagnosed with moderate-severe asthma and COPD [23,24]. Research studies have indicated that treatment with GFF leads to enhanced symptom management, HRQoL, and lung function compared to alternative therapies such as glycopyrronium/formoterol fumarate dihydrate [25]. Furthermore, the utilization of budesonide/formoterol/glycopyrronium (BDP/FF/GB) extra fine pressurized metered-dose inhaler (pMDI) over a duration of 26 weeks resulted in a significant enhancement in HRQoL, as assessed by the St. George Respiratory Questionnaire (SGRQ) and was deemed non-inferior to other triple therapies in COPD patients [24]. Moreover, the GFF combination has been consistently reported as well-tolerated, safe, and effective in enhancing HRQoL among individuals with these respiratory conditions [13,16]. As a result, GFF emerges as a valuable therapeutic option for patients managing these respiratory conditions.

The safety profile of glycopyrronium/formoterol combination therapy

Adverse Events

The safety profile of GFF therapy is generally favorable and well-tolerated among patients with COPD. However, certain adverse events (AEs) are associated with its use. Common side effects include dry mouth, nasopharyngitis, cough, bacterial upper respiratory tract infection (URTI), and headache [26]. Notably, significant cardiovascular side effects are similar to those observed with placebo and tiotropium [26]. The frequency of adverse events may vary depending on the study design and patient population. In a study comparing GFF combination therapy with glycopyrronium monotherapy, the most common AEs reported in the GFF group were headache (1.12%) and abnormal blood pressure (8.43%) [12]. Similarly, common AEs in another study included dry mouth, nasopharyngitis, cough, bacterial URTI, and headache [26]. In a randomized, seven-day study evaluating the efficacy and safety of a glycopyrrolate/formoterol fumarate fixed-dose combination metered dose inhaler (MDI), the incidence of treatment-emergent adverse events (TEAEs) was comparable for both doses of GFF MDI (31.7% vs. 27.9%) [26]. Furthermore, six serious adverse events (SAEs) were reported in five patients, none of which were deemed to be related to the study drug [26]. It is imperative to monitor patients for potential cardiovascular effects and hypokalemia while utilizing GFF therapy.

Cardiovascular Safety

Research into the cardiovascular safety of GFF therapy has been a focus of investigation. Studies have specifically examined the cardiovascular safety profile of fixed-dose combinations incorporating glycopyrronium and formoterol delivered via inhalers. These investigations have indicated that GFF therapy, administered through a metered dose inhaler utilizing Co-Suspension™ delivery technology, exhibits a favorable cardiovascular safety profile [27,28]. Additionally, the cardiovascular safety profiles of alternative combination therapies, such as QVA149 and budesonide/glycopyrrolate/formoterol fumarate, have also been evaluated, underscoring the importance of assessing cardiovascular effects when considering treatment options for respiratory conditions [29,30]. In summary, these findings suggest that GFF therapy generally poses minimal cardiovascular risks; however, diligent monitoring for potential effects remains crucial, particularly in patients diagnosed with COPD.

Respiratory Safety

Extensive research has thoroughly examined the respiratory safety profile of GFF combination therapy in individuals diagnosed with COPD. Findings from these studies suggest that GFF treatment significantly enhances lung function and quality of life while maintaining high patient tolerability [12,18]. Moreover, evidence indicates that GFF is safe and effective, with no discernible increase in safety risks compared to monotherapy or placebo. Notably, GFF therapy consistently yields superior lung function outcomes for patients grappling with COPD [31]. Furthermore, a randomized study evaluating the efficacy and safety of glycopyrrolate/formoterol fumarate fixed-dose combination, delivered via Co-Suspension™ technology, revealed promising results regarding both efficacy and safety among COPD patients [32]. These findings collectively underscore the favorable respiratory safety profile of GFF therapy, reinforcing its role as a valuable treatment option for individuals navigating the complexities of COPD.

Long-Term Safety Data

The long-term safety and efficacy of GFF therapy have been thoroughly investigated in patients grappling with moderate to severe COPD [33]. A pooled analysis of the PINNACLE studies underscored that GFF MDI 18/9.6 μg twice daily was well-tolerated and deemed safe for extended use among individuals diagnosed with COPD [17]. This comprehensive analysis further reaffirmed the sustained long-term safety and tolerability of GFF MDI 18/9.6 μg twice daily in subjects navigating moderate-to-very severe COPD [33]. Moreover, a study evaluating the cardiovascular safety profile of a fixed-dose combination comprising glycopyrrolate and formoterol fumarate, delivered via metered dose inhaler utilizing Co-Suspension™ delivery technology, concluded that the combination therapy was both safe and well-tolerated [28]. These findings collectively underscore the favorable long-term safety and tolerability profile of GFF therapy, reinforcing its suitability for extended use in patients managing moderate to severe COPD.

Comparative analysis with glycopyrronium monotherapy

Efficacy Comparison

The comparative analysis between glycopyrronium/formoterol fixed-dose combination (GB/FF) and glycopyrronium monotherapy in patients diagnosed with moderate-to-severe COPD has consistently demonstrated that GB/FF surpasses glycopyrronium monotherapy in terms of enhancing lung function and alleviating symptoms [13,14,34]. Furthermore, studies have indicated that GFF is non-inferior to TFF regarding improvements in lung function and health status assessment, with both treatments proving safe and well-tolerated for individuals experiencing symptomatic COPD [13,34]. The amassed evidence strongly supports utilizing the fixed-dose combination of glycopyrronium/formoterol as a beneficial treatment option for patients diagnosed with moderate-to-severe COPD, offering improved outcomes compared to glycopyrronium monotherapy.

Safety Comparison

The safety comparison between GB/FF and glycopyrronium monotherapy in patients diagnosed with moderate-to-severe COPD indicated that both treatments were well-tolerated and deemed safe for use [14]. Most AEs reported were of mild or moderate intensity, with low incidences observed in both treatment groups [14]. Additionally, clinically significant abnormalities in laboratory parameters, physical examinations, and vital signs were infrequent and were not attributed to the study drug [14]. Overall, the safety profile of GB/FF dry powder inhalation (DPI) and glycopyrronium monotherapy remained comparable across Indian patients and in global and Asian populations [14].

Cost-Effectiveness

The cost-effectiveness analysis comparing GFF to TFF in patients diagnosed with moderate-to-severe COPD indicated that GFF was non-inferior to TFF and exhibited better tolerability rendering it a viable alternative for individuals unable to tolerate TFF [13,35,36]. Research studies have further demonstrated that GFF yields significant improvements in lung function and health status assessment, positioning it as a beneficial option for the long-term management of COPD [13,36]. Moreover, the cost-effectiveness of fixed-dose combinations such as tiotropium/olodaterol (TIO/OLO) has been evaluated across various countries, revealing favorable outcomes for mitigating exacerbation risks and offering cost-effective treatment alternatives [37]. Overall, these findings underscore the importance of considering both efficacy and cost-effectiveness when selecting maintenance therapies for patients grappling with COPD.

Factors influencing treatment choice

Disease Severity

Comorbidities significantly heighten the risk of severe disease outcomes in patients. Conditions such as diabetes, hypertension, cardiovascular diseases, chronic kidney disease, and chronic liver disease have been identified as particularly concerning factors [38]. Specific medical conditions have also increased disease severity and mortality rates. Studies have shown that congestive heart failure, hilar lymphadenopathy, and bilateral lung involvement are among the medical conditions linked to heightened severity of illness [38]. In the context of COVID-19 in children, risk factors for disease severity have been identified. The WHO ordinal scale classification categories severity into five levels: uninfected to hospitalized moderate disease. This scale helps assess the severity of illness among pediatric patients with COVID-19 [39]. The severity of the illness can be further categorized into mild to moderate or severe based on specific risk factors for infection with particular pathogens and the timing of onset. Understanding these severity factors is crucial for accurately assessing and managing patients with various infectious diseases [40].

Patient Characteristics

Several factors influence treatment choices for patients, encompassing various aspects of their lives and health. Sociodemographic characteristics, including age, gender, and education level, play pivotal roles in treatment selection. Older patients may require different treatment options due to age-related physiological changes, while gender differences may affect medication responses. Moreover, education level can impact a patient's comprehension of treatment plans and adherence [41]. General health and clinical characteristics are key considerations in treatment decisions. The severity of symptoms, comorbidities, and overall health status directly influence treatment choices. Patients with severe symptoms may necessitate more aggressive treatment approaches, while those with multiple health conditions require comprehensive treatment plans addressing all aspects of their health [42].

Psychological characteristics and coping mechanisms are crucial determinants of treatment choice. Factors such as self-efficacy, mental health status, and coping strategies influence treatment adherence. Patients with higher self-efficacy are more likely to adhere to treatment regimens, whereas those with mental health issues may require tailored interventions and additional support [42]. Shared decision-making (SDM) style or preference is another factor in treatment selection. The extent to which patients wish to be involved in decision-making varies. Some prefer an active role, while others prefer a more passive approach, highlighting the importance of aligning treatment decisions with patient preferences [42].

Cultural and demographic factors, including ethnicity, race, and socioeconomic status, also shape treatment choices. Patients from diverse cultural backgrounds may hold distinct beliefs and preferences regarding treatment options. Additionally, socioeconomic disparities may limit access to specific treatments, emphasizing the need for culturally sensitive and equitable healthcare practices [43]. Patient preferences, such as treatment modality, convenience, and cost, significantly impact treatment decisions. Personal preferences regarding oral medication versus injections, convenience of treatment regimens, and financial considerations influence treatment selection [43]. Healthcare utilization patterns inform treatment decisions, including previous experiences with healthcare and primary care service utilization. Patients with differing healthcare utilization histories may possess distinct treatment expectations and preferences, underscoring the importance of considering individual healthcare experiences in treatment planning [43].

Physician Preferences

Physicians' preferences for medical innovation hold considerable sway over healthcare spending and the integration of novel medical procedures. Research indicates that physicians' inclinations significantly influence the adoption of new medical technologies within provider organizations, underscoring their pivotal role in the uptake of innovative treatments [44]. The reliance on physicians to determine appropriate patient treatments underscores the significance of understanding their social preferences. Health insurers and government agencies often lean on physicians in decision-making processes, emphasizing the importance of grasping their social preferences, which can profoundly impact treatment decisions and patient outcomes [45].

**Figure 1:**
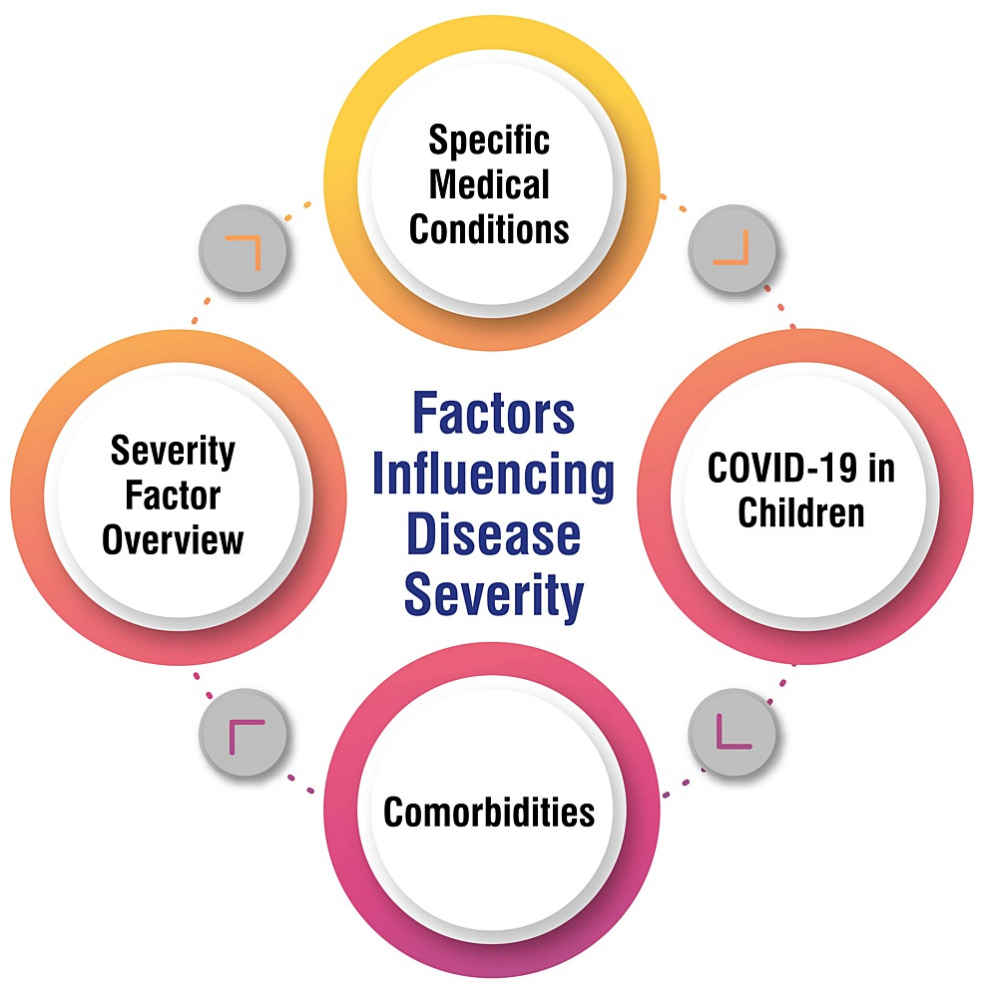
Factors influencing treatment choice Image credit: Bollineni S. Prada

Explorations into the disparities between physician and patient preferences for future therapies, such as in the realm of non-alcoholic steatohepatitis (NASH) treatments, have shed light on the necessity of understanding these preferences. Such understanding is paramount for developing effective treatment strategies that align with physician recommendations and patient needs [46]. Various demographic characteristics, including gender, age, income, education level, and health status, notably influence patients' preferences for their treating physician. Physicians are advised to cultivate trustful relationships with patients by being attentive listeners, providing informative discussions, maintaining eye contact, expressing interest, and demonstrating empathy [47]. Differences may arise between patient and physician priorities regarding treatment choices for newly diagnosed conditions. While patients may prioritize treatments that reduce hospitalization duration, physicians may place greater emphasis on treatments that enhance long-term outcomes, such as overall survival rates [47]. Figure 1 shows factors influencing treatment choice.

Future directions and unmet needs

Emerging Therapies

**Figure 2:**
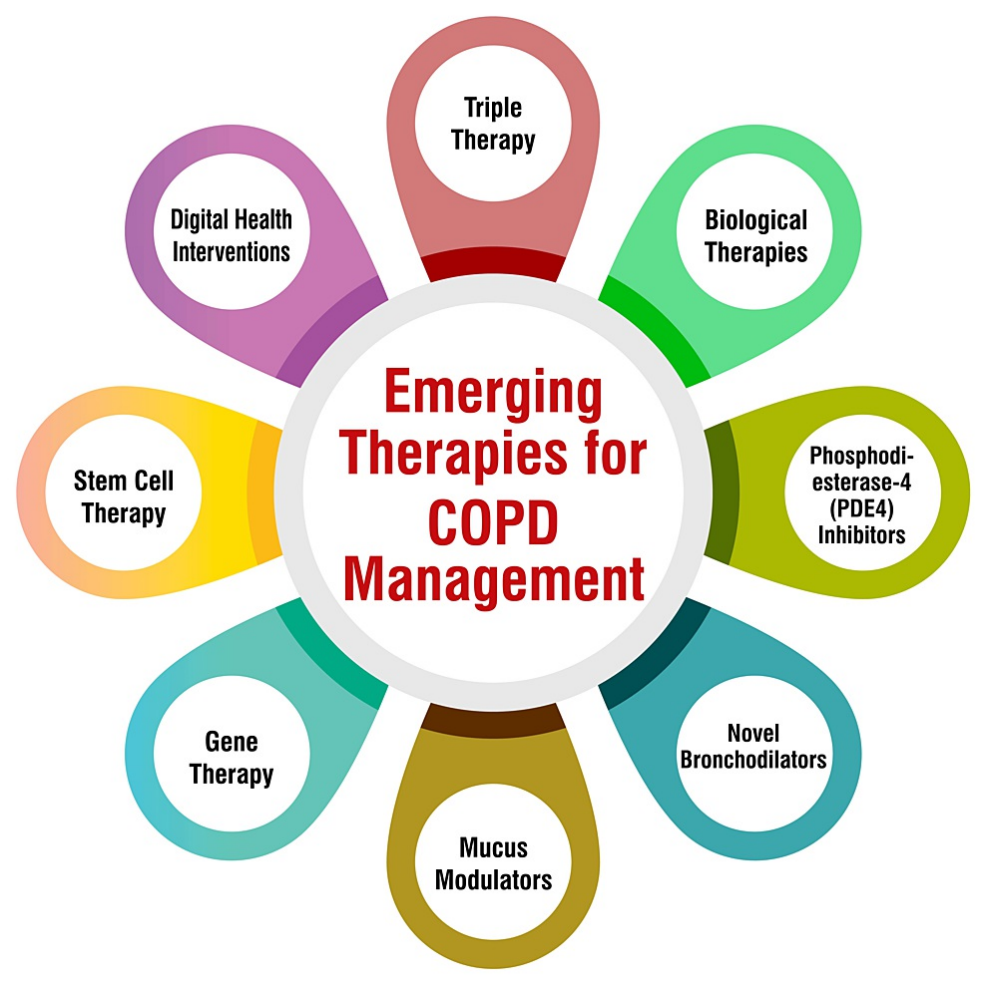
Emerging therapies for COPD management COPD: Chronic obstructive pulmonary disease Image credit: Bollineni S. Prada

Cell therapy and stem cell research advancements demonstrate promising potential for addressing various chronic diseases, including COPD. The establishment of the Novo Nordisk Foundation Center for Stem Cell Medicine (reNEW) marks a significant stride in this domain [48]. This collaborative effort among the University of Copenhagen in Denmark, Murdoch Children's Research Institute in Australia, and Leiden University Medical Center in The Netherlands aims to unlock the therapeutic capabilities of stem cells in organ development, tissue repair, and disease mechanisms [48]. The center will focus on three primary themes: rebuild, resolve, and rewriting, which will delve into leveraging stem cells to regenerate or reconstruct tissue, screen potential drug candidates, and devise novel treatment approaches for immune deficiency disorders and progressive congenital muscle disorders, respectively [48]. Moreover, the Novo Nordisk Foundation Cellerator, a cell therapy facility, has been established to bridge the chasm between cell therapy research and real-world treatments for chronic diseases such as Parkinson's, kidney disease, type 1 diabetes, and various forms of cancer [49]. The Cellerator will offer services encompassing process development, manufacturing, product release, and regulatory support, catering to public and private clients in academia, biotech, and the pharmaceutical industry [49]. These strides in cell therapy and stem cell research bring hope for forthcoming treatments and enhanced outcomes for patients grappling with chronic diseases, including COPD. Figure 2 shows emerging therapies for COPD management.

Patient-Centered Approaches

Patient-centered approaches to COPD care prioritize various aspects to ensure comprehensive and effective condition management. Central to this approach is patient education, which involves informing patients about their condition, involving them in clinical decisions, understanding their medication preferences, and addressing their emotional and physical needs and those of their families or caregivers [50]. Treatment adherence is critical in COPD management, and factors such as patient beliefs about medications, inhaler technique, and choice of delivery devices significantly impact therapeutic success [50]. Enhancing transitions of care across healthcare settings is also crucial for ensuring coordination and continuity of care for COPD patients [51]. Developing patient-centered discharge care bundles based on evidence, multidisciplinary consensus, and patient input can improve COPD management by addressing critical aspects such as inhaler technique demonstration, medication optimization, referral to pulmonary rehabilitation, and smoking cessation support [51]. Furthermore, integrating comorbidities and phenotype-based medicine into patient-centered COPD care can optimize treatment outcomes by addressing individual needs and tailoring therapies accordingly [52]. The Patient-Centered Medical Home (PCMH) model has emerged as a promising framework for transforming primary care in COPD management, emphasizing proactive, team-based care focused on maintenance rather than acute rescue [53]. Additionally, structured and systematic team-based follow-up interventions, such as Guided Self-Determination, have shown potential benefits for COPD patients by supporting self-management skills and improving overall care outcomes [54]. These approaches aim to enhance patient empowerment, improve treatment adherence, and optimize outcomes for individuals with COPD.

## Conclusions

Comparing the combination therapy of glycopyrronium/formoterol to glycopyrronium monotherapy in moderate-to-severe COPD reveals clear benefits for combination therapy. It consistently shows better lung function, symptom control, and fewer exacerbations. The safety profile also seems favorable. This suggests combination therapy is preferable for patients with persistent symptoms despite prior treatment. Healthcare providers should consider personalized treatment plans with combination therapy. Regular monitoring is crucial, and more research is needed for long-term safety and cost-effectiveness. Adhering to evidence-based practices can improve COPD management and patient outcomes.
